# Combined Effect of Health Status and Primary Care Use on Participation in Cancer Screening: The CONSTANCES Cohort

**DOI:** 10.1089/whr.2020.0096

**Published:** 2020-10-28

**Authors:** Thi-Van-Trinh Tran, Jeanna-Eve Franck, Mireille Cœuret-Pellicer, Laurent Rigal, Virginie Ringa, Gwenn Menvielle

**Affiliations:** ^1^ Sorbonne Université, Inserm, Institut Pierre Louis d'Epidémiologie et de Santé Publique (IPLESP), Paris, France.; ^2^ Inserm-Versailles Saint Quentinen Yvelines University, UMS 011 “Epidemiological Population-Based Cohorts Unit,” Villejuif, France.; ^3^ CESP Centre for Research in Epidemiology and Population Health, U1018, Gender, Sexuality and Health Team, University of Paris-Saclay, University of Paris-Sud, UVSQ, Villejuif, France.; ^4^ Ined, Paris, France.

**Keywords:** breast cancer screening, cervical cancer screening, health status, primary care utilization

## Abstract

**Background:**

The combined association between primary care utilization and health status with breast cancer screening (BCS) and cervical cancer screening (CCS) remains unclear. Our aim was to identify women's profiles based on their health status and primary care utilization and study their associated adherence to BCS and CCS recommendations.

**Methods:**

Using data from the cohort of people visiting health screening centers (CONSTANCES) in France (2012–2015), we first identified women's profiles based on their health status (self-perceived health, physical, and mental health) and primary care utilization (visit to the General Practitioner [GP], uptake of blood tests) using a multiple correspondence analysis and a hierarchical cluster analysis. We then investigated the association of these profiles to BCS and CCS using logistic regression models adjusted for age, smoking status, sociodemographic and socioeconomic characteristics, and the regularity of gynecologist consultation.

**Results:**

We identified five distinct profiles of women with contrasted participation in BCS (*n* = 14,122) and CCS (*n* = 27,120). In multivariate analyses, cancer screening participation increased from women with very good health and poor primary care utilization, to those with poor health and frequent visits to the GP, and those with very good health and average primary care utilization. The most favorable profiles regarding cancer screening rates were women with average-to-poor health and regular visits to the GP and uptake of blood tests.

**Conclusions:**

Our results suggest that policies aiming at increasing cancer screening participation should simultaneously account for women's use of primary care and health and consider more specific subgroups than what is usually done. Further research should investigate factors motivating cancer screening practice, such as women's beliefs regarding cancer screening and women's psychological characteristics.

## Introduction

Breast cancer screening (BCS) and cervical cancer screening (CCS) are efficient tools to reduce the burden of these diseases. However, actual BCS and CCS rates in France are still far from the goal of 80% established by the French public health law of 2004 (about 65% and 60% in the 2010s, respectively).

Since 2004, BCS has been proposed free of charge in a national organized program to women 50–74 years of age every 2 years. This program exists alongside opportunistic screening. CCS is recommended every 3 years for women 25–65 years of age. With the exception of some local organized programs, CCS was mainly opportunistic until 2020. A nationwide organized CCS program is being implemented in 2020. CCS is mostly performed by gynecologists but can also be performed by general practitioners (GP) or midwifes. In addition, GPs are financially encouraged to ensure their patients have BCS and CCS on time.

Along with sociodemographic and socioeconomic circumstances, health behaviors, and health care access,^1,2^ primary care utilization is likely to hold a close association with participation in BCS and CCS. Accumulating evidence suggests that GPs play an important role in encouraging their patients to have on time BCS and CCS.^3–5^ The number of visits to a GP, the regularity of GP consultation, and also the uptake of other general preventive exams are potential factors affecting adherence to cancer screening recommendations.^2,6–10^

The association between participation in BCS or CCS and primary care utilization can be markedly influenced by women's health status. Prior studies have consistently reported that health indices such as a high body mass index (BMI), the presence of physical or mental chronic conditions, as well as poor self-perceived health were associated with a lower adherence to BCS and CCS recommendations^11–16^ and with an increased primary care utilization.^17–19^ In addition, increased primary care utilization is associated with a higher adherence to BCS and CCS recommendations.^6,12^

On the other hand, women with good health tend to visit less frequently their GP but have high rates of BCS and CCS. The combined association of primary care utilization and health status with cancer screening is then complex, and multiple adjustments only provide a partial understanding. A better understanding would allow identifying more precisely the groups that do not meet screening recommendations and would help target the groups most at risk for nonparticipation in cancer screening. This would improve cancer screening rates, which is a public health goal.

To provide new insights into the intertwined effect of health status and primary care utilization on cancer screening, we chose to identify women's profiles accounting for their health status and primary care utilization simultaneously, and study the association between these profiles and women's adherence to BCS and CCS recommendations.

## Materials and Methods

The cohort of people visiting health screening centers (CONSTANCES) was approved by the national authority on data protection. CONSTANCES is a prospective study of French adults 18–69 years of age at inception. Participants are randomly selected from adults covered by the National Health Insurance Fund. They were invited by mail for a health examination in 22 selected health screening centers throughout France. The response rate was about 10%, which is similar to other large cohorts.^20^ At inclusion, sociodemographic, socioeconomic, and health-related data are collected through questionnaires and during a health examination.^21^

The present study included women recruited in CONSTANCES from 2012 to 2015. Our analysis is based on data collected at inclusion augmented with exhaustive information on individual health care use (including cancer screening and medical visit) extracted from the national health insurance database for the 3 years before inclusion. The CONSTANCES cohort and the present study are approved by the national administrative authority on data protection.

The sample used to study BCS included women 50–74 years of age. We excluded women with personal or parental history of breast or ovarian cancer, or personal history of Hodgkin's lymphoma. In all, 14,122 women were eligible. The sample used to study CCS included women 25–65 years of age. We excluded women with hysterectomy, personal history of cervical or uterine cancer, or no sexual intercourse. In all, 27,120 women were eligible. Our outcome was having a BCS during the 2 years before inclusion (yes/no) or having a CCS during the 3 years before inclusion (yes/no).

In a first step, we identified the profiles of women based on their health status and primary care utilization. To do so, we conducted a hierarchical cluster analysis after a multiple correspondence analysis.^22^ The analysis was based on the following variables (categories of the variables are presented in Tables 1 and 2):

**Table 2.** Description of the Five Profiles of Women Combining Health and Primary Care Utilization Characteristics in the Cervical Cancer Screening Population

	All women		Profile A	Profile B	Profile C	Profile D	Profile E
	N (%)	Screening rate	%	%	%	%	%
All women	23,541 (100.0)	79.4					
Profile A	4,960 (21.1)	70.8					
Profile B	7,846 (33.3)	81.7					
Profile C	5,237 (22.2)	84.1					
Profile D	3,595 (15.3)	82.6					
Profile E	1,903 (8.1)	72.8					
Health status							
Self-perceived health							
Good to very good	18,658 (79.3)	80.7	86.2	91.6	83.4	63.5	28.6
Fair	4,040 (17.2)	74.8	11.5	7.1	14.8	30.0	55.5
Poor to very bad	843 (3.6)	72.1	2.3	1.2	1.8	6.6	15.9
Measured BMI							
Obesity	2,540 (10.8)	69.0	7.3	6.4	9.3	18.6	27.1
Overweight	5,243 (22.3)	76.2	21.7	18.7	24.7	25.6	25.7
Normal weight	14,831 (63.0)	82.0	70.9	65.9	65.7	51.5	44.9
Underweight	927 (3.9)	82.5	0.2	8.9	0.3	4.3	2.3
Long-term illness fee exemption							
No	21,533 (91.5)	79.6	95.9	97.4	98.3	76.9	64.2
Yes	2,008 (8.5)	76.7	4.1	2.6	1.7	23.1	35.8
At least one comorbidity							
No	19,646 (83.5)	79.8	92.5	91.7	85.8	62.8	58.3
Yes	3,895 (16.5)	77.2	7.5	8.3	14.2	37.2	41.7
Self-reported physical limitation							
No	20,011 (85.0)	80.3	89.3	94.2	89.1	73.8	45.8
Yes	3,530 (15.0)	73.7	10.7	5.8	10.9	26.2	54.2
Self-reported cognitive limitation							
No	20,664 (87.8)	80.3	88.7	92.7	90.5	84.9	63.0
Yes	2,877 (12.2)	72.4	11.3	7.3	9.5	15.1	37.0
Self-reported depression							
No	19,195 (81.5)	80.3	86.1	90.0	83.2	74.7	43.1
Yes	4,346 (18.5)	75.1	13.9	10.0	16.8	25.3	56.9
Primary care utilization							
No. of visits to a GP in the inclusion year							
0	2,187 (9.3)	70.1	17.7	10.9	5.7	2.7	2.9
1–3	9,794 (41.6)	79.2	50.3	49.2	39.3	25.7	23.6
4–6	6,621 (28.1)	81.6	21.7	26.8	33.6	32.2	27.6
>6	4,939 (21.0)	80.8	10.2	13.1	21.3	39.4	45.9
Regularity of glucose test during the last 3 years							
None	6,066 (25.8)	72.1	99.3	10.7	2.1	2.0	6.3
At least once a year one of the 3 years	8,861 (37.6)	80.6	0.6	87.5	10.9	6.7	60.6
At least once a year two of the 3 years	5,883 (25.0)	83.2	0.0	1.8	87.0	15.5	33.1
At least once a year each of the 3 years	2,731 (11.6)	83.0	0.0	0.0	0.0	75.9	0.1
Regularity of creatinine test during the last 3 years							
None	8,902 (37.8)	77.1	99.5	36.5	15.6	5.0	5.9
At least once a year one of the 3 years	7,873 (33.4)	80.8	0.5	62.4	26.3	11.7	61.0
At least once a year two of the 3 years	4,408 (18.7)	80.4	0.1	1.2	58.1	17.9	32.9
At least once a year each of the 3 years	2,358 (10.0)	80.9	0.0	0.0	0.0	65.5	0.2
Other characteristics							
Age							
25–34	5,079 (21.6)	77.8	23.0	26.5	20.5	14.2	14.0
35–44	6,299 (26.8)	82.3	33.3	29.1	24.2	19.3	21.1
45–54	6,324 (26.9)	81.8	27.4	25.4	28.1	24.6	32.4
55–65	5,839 (24.8)	74.9	16.2	19.0	27.1	41.9	32.5
Composition of household							
Couple with children	10,565 (44.9)	83.4	49.9	47.9	44.8	35.9	36.4
Couple without children	5,927 (25.2)	78.1	21.7	24.4	26.0	31.0	24.1
Single adult with children	2,032 (8.6)	79.0	8.5	7.9	8.4	8.3	13.4
Single adult without children	3,696 (15.7)	72.0	16.0	14.7	15.0	17.6	17.5
MD	1,321 (5.6)	73.5	3.9	5.1	5.7	7.3	8.7
Migration origin							
French with two French parents	18,893 (80.3)	80.2	80.2	82.4	80.4	78.2	75.2
French with at least one foreign parent	2,643 (11.2)	77.3	11.1	10.6	11.6	11.7	12.0
Naturalized immigrant	949 (4.0)	77.9	3.3	3.1	4.2	6.0	5.4
Foreign immigrant	723 (3.1)	69.2	4.3	2.8	2.3	2.2	4.6
MD	333 (1.4)	75.7	1.1	1.0	1.5	1.9	2.8
Occupational class							
Higher level professionals and managers	5,884 (25.0)	81.2	27.2	28.4	24.9	20.1	14.9
Lower level professionals	7,729 (32.8)	81.3	33.5	34.5	33.4	31.5	25.0
Clerical, sales and service	8,184 (34.8)	77.8	32.9	31.3	34.7	39.1	46.2
Laborers and factory workers	850 (3.6)	69.9	2.7	2.5	3.4	5.1	7.9
Self-employed and entrepreneurs	268 (1.1)	74.6	1.4	0.9	1.1	1.2	1.2
Never worked	278 (1.2)	66.9	1.0	1.0	1.0	1.4	2.3
MD	348 (1.5)	77.6	1.2	1.4	1.4	1.6	2.6
Financial difficulties							
Never	13,861 (58.9)	81.9	60.1	64.3	59.9	54.4	38.9
Occurred in the past	6,108 (25.9)	77.5	25.0	24.3	25.9	27.8	31.7
Yes	3,312 (14.1)	72.4	14.0	10.5	13.0	16.4	27.6
MD	260 (1.1)	76.2	0.8	0.9	1.2	1.4	1.7
Free health insurance for low income							
No	23,075 (98.0)	79.6	98.2	98.6	98.6	97.4	94.8
Yes	466 (2.0)	65.5	1.8	1.4	1.4	2.6	5.2
Smoking status							
Smoker	4,594 (19.5)	75.1	22.1	20.0	18.0	15.2	23.2
Ex-smoker	7,007 (29.8)	81.4	27.6	29.3	31.5	31.8	28.5
Never-smoker	11,017 (46.8)	79.9	46.3	47.0	47.0	48.5	43.4
MD	923 (3.9)	78.0	4.0	3.7	3.4	4.5	5.0
Regularity of gynecologist consultation during the last 3 years							
None	5,332 (22.6)	43.9	29.5	20.2	18.2	21.2	29.8
At least once a year one of the 3 years	4,443 (18.9)	79.4	22.0	18.2	17.3	16.7	21.9
At least once a year two of the 3 years	5,718 (24.3)	90.4	23.9	25.4	24.5	23.3	22.0
At least once a year each of the 3 years	8,048 (34.2)	95.0	24.6	36.2	40.0	38.7	26.3

Constances cohort, France, 2012–2015 (*n* = 23,541). The population presented in the table is the population used in the multiple correspondence analysis and cluster analysis.

MD, missing data.

**Table 1.** Description of the Five Profiles of Women Combining Health and Primary Care Utilization Characteristics in the Breast Cancer Screening Population

	All women		Profile A	Profile B	Profile C	Profile D	Profile E
	N (%)	Screening rate	%	%	%	%	%
All women	11,491 (100.0)	82.1					
Profile A	2,583 (22.5)	77.1					
Profile B	2,143 (18.6)	82.0					
Profile C	2,703 (23.5)	86.1					
Profile D	2,742 (23.9)	85.4					
Profile E	1,320 (11.5)	77.3					
Health status							
Self-perceived health							
Good to very good	8,473 (73.7)	82.7	91.1	89.9	80.2	61.6	25.6
Fair	2,550 (22.2)	80.8	7.9	9.1	18.8	33.9	54.0
Poor to very bad	468 (4.1)	79.3	1.0	1.0	1.0	4.5	20.4
Measured BMI							
Obesity	1,613 (14.0)	77.5	6.6	6.7	13.6	24.4	19.8
Overweight	3,222 (28.0)	83.1	24.4	26.5	27.5	28.0	38.9
Normal weight	6,387 (55.6)	82.9	66.4	63.2	58.0	45.7	37.9
Underweight	269 (2.3)	80.7	2.6	3.6	1.0	1.9	3.3
Long-term illness fee exemption							
No	10,145 (88.3)	82.4	96.4	96.9	92.1	76.3	75.5
Yes	1,346 (11.7)	80.0	3.6	3.1	7.9	23.7	24.5
At least one comorbidity							
No	7,649 (66.6)	80.9	83.6	81.1	63.4	45.0	60.9
Yes	3,842 (33.4)	84.5	16.4	18.9	36.6	55.0	39.1
Self-reported physical limitation							
No	8,938 (77.8)	82.7	91.0	90.4	84.5	70.1	33.6
Yes	2,553 (22.2)	80.1	9.0	9.6	15.5	29.9	66.4
Self-reported cognitive limitation							
No	9,928 (86.4)	82.7	92.6	90.9	92.3	87.2	52.9
Yes	1,563 (13.6)	78.1	7.4	9.1	7.7	12.8	47.1
Self-reported depression							
No	9,357 (81.4)	82.8	92.0	90.6	86.8	79.6	38.6
Yes	2,134 (18.6)	79.1	8.0	9.4	13.2	20.4	61.4
Primary care utilization							
No. of visits to a GP in the inclusion year							
0	816 (7.1)	70.8	19.4	9.3	1.6	1.3	2.8
1–3	4,127 (35.9)	81.0	46.6	47.3	35.0	23.5	24.0
4–6	3,618 (31.5)	84.0	23.5	30.2	37.2	34.8	30.5
>6	2,930 (25.5)	84.5	10.4	13.2	26.2	40.3	42.7
Regularity of glucose test during the last 3 years							
None	2,099 (18.3)	73.2	67.5	0.0	0.0	1.2	24.5
At least once a year one of the 3 years	3,802 (33.1)	82.5	21.6	99.6	12.2	9.5	39.2
At least once a year two of the 3 years	3,446 (30.0)	84.9	10.1	0.4	87.8	16.2	27.1
At least once a year each of the 3 years	2,144 (18.7)	85.6	0.7	0.0	0.0	73.1	9.2
Regularity of creatinine tests during the last 3 years							
None	2,540 (22.1)	76.3	84.1	0.0	0.0	2.0	23.7
At least once a year one of the 3 years	3,632 (31.6)	82.1	10.8	99.7	18.1	8.8	37.0
At least once a year two of the 3 years	3,143 (27.4)	85.0	4.8	0.3	81.9	15.4	28.4
At least once a year each of the 3 years	2,176 (18.9)	84.8	0.3	0.0	0.0	73.9	10.9
Other characteristics							
Age							
50–54	3,138 (27.3)	78.2	34.6	31.3	24.9	18.5	29.8
55–59	3,091 (26.9)	81.3	29.1	28.7	24.7	24.5	29.2
60–64	2,917 (25.4)	84.3	22.8	24.2	27.1	29.1	21.2
65–69	2,345 (20.4)	85.7	13.5	15.9	23.3	27.9	19.8
Composition of household							
Couple with children	2,359 (20.5)	79.7	26.1	22.7	18.1	16.1	20.3
Couple without children	4,819 (41.9)	84.8	39.3	41.5	45.8	44.6	34.3
Single adult with children	885 (7.7)	78.0	8.4	7.8	6.5	6.4	11.4
Single adult without children	2,223 (19.3)	81.0	18.5	17.8	18.4	21.2	21.6
MD	1,205 (10.5)	81.4	7.7	10.1	11.2	11.7	12.4
Migration origin							
French with two French parents	9,321 (81.1)	82.9	83.0	83.3	82.9	79.9	72.9
French with at least one foreign parent	1,175 (10.2)	79.3	9.7	9.3	9.8	11.3	11.4
Naturalized immigrant	538 (4.7)	78.8	3.7	3.9	4.1	5.4	7.7
Foreign immigrant	220 (1.9)	74.5	2.3	1.8	1.1	1.3	4.2
MD	237 (2.1)	80.6	1.3	1.7	2.1	2.2	3.8
Occupational class							
Higher level professionals and managers	2,515 (21.9)	81.1	28.8	24.1	21.0	18.5	13.6
Lower level professionals	4,103 (35.7)	83.7	38.1	38.8	36.5	33.9	28.3
Clerical, sales, and service	3,784 (32.9)	82.0	26.6	30.1	33.2	36.9	41.0
Laborers and factory workers	542 (4.7)	81.4	2.8	3.4	4.9	5.3	8.9
Self-employed and entrepreneurs	183 (1.6)	77.0	1.4	1.3	1.7	1.9	1.7
Never worked	156 (1.4)	80.1	0.8	1.0	1.1	1.6	3.2
MD	208 (1.8)	73.1	1.4	1.3	1.6	2.0	3.3
Financial difficulties							
Never	7,055 (61.4)	84.4	67.7	66.4	64.4	59.0	39.8
Occurred in the past	3,095 (26.9)	80.5	24.5	25.6	26.0	27.9	33.9
Yes	1,175 (10.2)	72.9	6.7	6.7	8.3	11.6	23.8
MD	166 (1.4)	80.7	1.1	1.3	1.3	1.5	2.5
Free health insurance for low income							
No	11,363 (98.9)	82.4	99.5	99.0	99.4	98.8	96.6
Yes	128 (1.1)	57.0	0.5	1.0	0.6	1.2	3.4
Smoking status							
Smoker	1,418 (12.3)	74.5	13.2	13.3	11.4	9.7	16.7
Ex-smoker	3,952 (34.4)	82.7	35.3	35.4	34.4	34.3	31.2
Never-smoker	5,626 (49.0)	83.9	47.3	47.2	49.9	51.7	47.5
MD	495 (4.3)	79.8	4.2	4.2	4.3	4.3	4.6
Regularity of gynecologist consultation during the last 3 years							
None	3,600 (31.3)	72.2	31.7	29.1	29.5	31.0	38.6
At least once a year one of the 3 years	2,338 (20.3)	79.6	19.5	21.4	20.6	19.3	22.0
At least once a year two of the 3 years	2,471 (21.5)	86.7	21.4	22.2	21.4	22.0	19.8
At least once a year each of the 3 years	3,082 (26.8)	91.9	27.4	27.3	28.5	27.7	19.5

Constances cohort France, 2012–2015 (*n* = 11,491). The population presented in the table is the population used in the multiple correspondence analysis and cluster analysis.

BMI, body mass index; GP, general practitioner; MD, missing data.

- Primary care utilization: number of visits to a GP during the inclusion year, regularity of taking glucose and creatinine tests over the 3 years before inclusion.
- Health status: self-perceived health, measured BMI, having at least one comorbidity (self-reported cardiovascular, respiratory, or osteoarticular diseases, treated diabetes, treated hypercholesterolemia or hypertriglyceridemia, history of cancer), long-term illness fee exemption, self-reported depression (using the CESD scale), self-reported physical limitation (at least one limitation among the following: difficulties in walking up/down stairs, walking 1 km without stopping or carrying 5 kg for 10 m), and self-reported cognitive limitation (at least one limitation among the following: difficulties in reading, writing, or calculating or needing help in administrative work).

Missing data were below 5% for all variables except depression (5.5% for CCS, 9.7% for BCS). Only women without missing data were included (*N* = 11,491 for BCS and *N* = 23,541 for CCS). The optimal number of clusters was determined with cluster dendrogram, cubic clustering criterion, pseudo-F, and silhouette. The identified clusters were then described according to the following variables: primary care utilization and health status, but also sociodemographic characteristics (age, composition of the household, migration origin), socioeconomic factors (occupational class, financial difficulties, free health insurance for low income), smoking status, and regularity of gynecologist consultations over the 3 years before inclusion (categories of the variables are presented in Tables 1 and 2).

In a second step, the association between on-time cancer screening and these clusters was examined with logistic regression models. Five models were performed: nonadjusted; adjusted for age; adjusted for age, smoking status, demographic, and socioeconomic characteristics; adjusted for age and the regularity of gynecologist consultations; and adjusted for all covariates. Missing data were below 5% for all adjustment variables except the composition of the household (Tables 1 and 2). Only women without missing data in all covariates were included in the logistic regression models (*N* = 9,435 for BCS and *N* = 20,603 for CCS).

Statistical analyses were carried out with SAS version 9.4 and the FactoMineR^23^ and NbClust^24^ packages of R version 3.4.3.

## Results

In our data, 82.1% and 79.4% of women had BCS and CCS on time, respectively (Tables 1 and 2). Nine out of 10 women visited a GP at least once in the inclusion year, over 75% undertook glucose and creatinine test at least once during the 3 years before inclusion. About 75% of the women rated their health as good or very good. More than half had a BMI below 25 and more than two third did not report any comorbidity, cognitive or physical limitations, or depression.

**FIG. 1.**
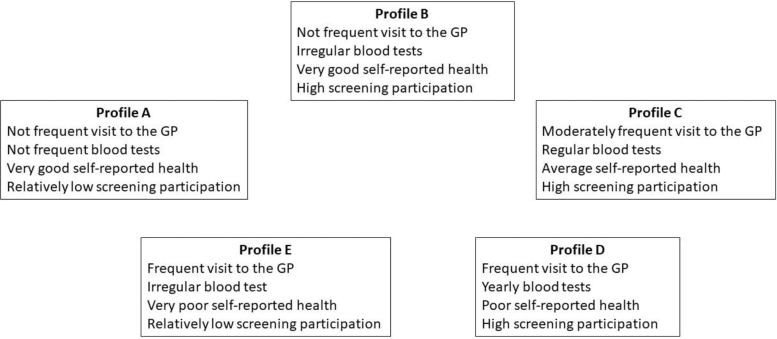
Qualitative description of the five profiles combining primary care utilization and health status. GP, general practitioner.

The clustering procedure resulted in the identification of five distinct clusters (hereafter called profiles A to E) for both populations. The five profiles are described in Tables 1 and 2. The distribution of the women across the five profiles A to E is as follows: 22.5%, 18.6%, 23.5%, 23.9%, and 11.5% in the BCS population; 21.1%, 33.3%, 22.2%, 15.3%, and 8.1% in the CCS population. The key characteristics of the profiles are similar for the BCS and CCS populations. They are summarized in Figure 1 and detailed below.

Compared with women in the other profiles, women in profile A the least frequently visited a GP. Most of them did not have any blood tests during the 3 years before inclusion. From profile B to profile D, the number of visits to a GP in the inclusion year increased gradually parallel to the regularity of blood tests. Most women in profile B had one blood test during one of the 3 years before inclusion, whereas most women in profile D had one blood test each of the 3 years before inclusion. The number of visits to a GP during the inclusion year of women in profile E was similar to that of women in profile D, while their regularity of blood tests was markedly lower.

Women in profiles A and B had very good health. Then, the health status decreased progressively from profile B to profile E.

Age increased progressively from profile A to profile D. Women in profile E tended to be younger than the other women in the BCS population and older in the CCS population. Women in profiles A and B had close and high socioeconomic position, then the socioeconomic position decreased progressively from profile B to profile E. The follow-up by a gynecologist was frequent and similar in the profiles B, C, and D, and then progressively decreased in profile A and in profile E. Noteworthy, profiles A and E had similar follow-up in the CCS population.

Table 3 shows the crude screening rates and the odds ratios (ORs) for participation in BCS and CCS associated with the five profiles. The screening rate was lower for women in Profile A (77.1% for BCS, 71.2% for CCS) and profile E (78.3% for BCS, 73.4% for CCS) compared with other women (81.8%–86.1% for BCS, 82.1%–84.6% for CCS).

**Table 3.** Odds Ratios for Participation in Breast and Cervical Cancer Screening According to the Five Profiles of Women Combining Health and Primary Care Utilization

			Nonadjusted		Adjusted for							
					Age		Smoking status, sociodemographic and socioeconomic characteristics^a^		Age and the regularity of gynecologist consultation		All covariates	
Women's profile	N	Screening rate	OR	95% CI	OR	95% CI	OR	95% CI	OR	95% CI	OR	95% CI
Breast cancer screening	9,435											
Profile A	2,218	77.1	0.52	0.45–0.61	0.55	0.47–0.64	0.55	0.46–0.64	0.56	0.48–0.66	0.57	0.48–0.67
Profile B	1,773	81.8	0.70	0.59–0.83	0.73	0.61–0.86	0.72	0.61–0.86	0.73	0.61–0.87	0.73	0.62–0.88
Profile C	2,197	86.5	1		1		1		1		1	
Profile D	2,223	86.1	0.97	0.82–1.15	0.94	0.80–1.12	0.98	0.82–1.16	0.93	0.78–1.11	0.95	0.80–1.14
Profile E	1,024	78.3	0.56	0.46–0.61	0.58	0.48–0.70	0.66	0.54–0.81	0.67	0.55–0.81	0.74	0.60–0.90
Cervical cancer screening	20,603											
Profile A	4,450	71.2	0.45	0.41–0.50	0.43	0.39–0.47	0.43	0.38–0.47	0.58	0.52–0.65	0.58	0.52–0.66
Profile B	6,941	82.1	0.84	0.76–0.93	0.81	0.73–0.90	0.78	0.71–0.87	0.89	0.79–0.99	0.87	0.77–0.98
Profile C	4,596	84.6	1		1		1		1		1	
Profile D	3,061	83.3	0.91	0.80–1.03	0.97	0.85–1.10	1.02	0.90–1.16	1.00	0.87–1.16	1.04	0.90–1.20
Profile E	1,555	73.4	0.51	0.44–0.58	0.51	0.44–0.58	0.59	0.51–0.68	0.66	0.57–0.78	0.72	0.61–0.85

Constances cohort, France, 2012–2015.

^a^ Adjusted for age, composition of household, migration origin, financial difficulties, free health insurance for low income, occupational class, and smoking status.

CI, confidence interval.

In the logistic regression models, women in profile C were used as the reference category. In the BCS population, the various adjustments did not largely modify the ORs, except for women in profile E where the OR increased in the fully adjusted model. In the CCS population, odds for on-time screening increased after adjustment for age, smoking status, sociodemographic, and socioeconomic variables for women in profile E. On the contrary, adjustment for age and the regularity of visits to a gynecologist reduced the odds for on-time screening for women in profiles A and E. In the fully adjusted model, a gradient was observed for participation in BCS and CCS, ranging from profile A, to profile E, profile B, and then profiles C and D, the two latter being not statistically different.

## Discussion

In this large national sample, we identified five profiles of women based on their primary care utilization and health status with contrasted adherence to BCS and CCS recommendations. Women with the best health, and the highest socioeconomic position, do not necessarily have the best screening practices. A considerable number of healthy women with favorable socioeconomic circumstances but poor primary care utilization are unlikely to have BCS or CCS on time and even have the worst screening practice in our data. By contrast, some of the unhealthy women with very good primary care utilization still adhere to cancer screening recommendations.

The number of yearly visits to the GP and the regularity of blood tests were used to characterize the primary care utilization. The link between primary care utilization and cancer screening may result from the health care follow-up but also from women's characteristics. Indeed, it is possible that from a certain level of visits to the GP, women had more chance to be checked up and to receive preventive care.^25^ A low uptake of blood tests may happen because the GP did not prescribe the test (lack of time among healthy women who very rarely visit the GP or competing health concerns among women suffering from comorbidities^26,27^).

It is also possible that the women did not adhere to the prescription. Studies indeed reported that lower adherence to prescription was associated to numerous factors such as age, health, health perceptions, or socioeconomic conditions.^28,29^ Therefore, the regularity of blood tests could be interpreted as a marker of the intensity and quality of the women's medical follow-up. However, if this would apply to women in poor health, among whom regular tests are required, this is not the case among healthy women. The regularity of blood tests may be a proxy of the woman's need to be reassured regarding her health, but also reflects her incorporation of a medical norm.

Consistently with previous research, we found that primary care utilization increased as health decreased.^16–18^ However, we observed that this did not necessarily impact BCS and CCS practice. We identified three groups of women (profiles B, C, and D) that strongly differed regarding health status and primary care utilization (decreasing health associated with increasing primary care utilization) but had high BCS and CCS crude rates. Noteworthy, among these three profiles, women with the best health condition and the poorest primary care utilization (profile B) were the least likely to have cancer screening on time. In addition to the effect of primary care utilization discussed above, worse health might make women pay more attention to their health^19^ as well as to choose a healthy lifestyle, including undergoing cancer screening.

Prior studies suggested that poor health was strongly associated with a low adherence to BCS and CCS recommendations.^11,15,16^ Consistently with the literature, we identified a group of women having poor health and low cancer screening rates (profile E). However, we also identified a group of women with impaired health but high screening rates (profile D). Compared with women in profile D, women in profile E differ in several aspects that may explain their lower cancer screening participation: they had a substantially poorer health (they were more frequently obese, reported poorer mental health, and more limitations) and experienced more extreme socioeconomic situations and in particular were markedly more frequently single mothers.

This accumulated effect could lead to a much higher level of financial hardship, more difficulties for attending medical appointments, and a heavier burden of health on women's life. As a consequence, in profile E, health care, and in particular prevention, might not be these women's first priority, but earning their living.^30^ In addition, among women in profile E, more pronounced financial hardship may be a barrier to visit to a gynecologist (which implies an on average large out-of-pocket payment). Moreover, because of these women's poorer health status, GPs may focus more on curative care than preventive care, which may account for both their lower BCS and CCS rates as well as their lower regularity of blood tests.^26,27^ Regarding the latter, it is also possible that these women did not perform the prescribed blood test due to the accumulated effect of unfavorable conditions.^28,29^

Despite a few dissenting findings,^31^ most studies showed that good health was associated with an improved adherence to cancer screening recommendations.^1,2^ Consistently with the literature, we identified one profile with good health and high BCS and CCS rates (profile B). However, we also identified a group of women in the best health condition but with low cancer screening rates (profile A). Both profiles had favorable socioeconomic position; they did not differ on social support level, proxied by the composition of the household, or rural/urban place of residence (results not shown), factors related to the adherence to cancer screening.^1,2,9^

The different participation in cancer screening for the two groups may be explained by differences in personality traits, namely women's perception, beliefs, and attitudes toward cancer screening.^32–36^ Fear and perceived danger might be factors either facilitating or preventing the screening practice.^37^ People who do not attend screening are likely to express either great distress or a feeling of not being concerned while the others have screening to reassure themselves.^37,38^ This feeling of not being concerned by cancer screening may be reinforced in healthy women who might not consider themselves to be threatened by illness, including breast and cervical cancer, nor to be in the target group of cancer screening.^9,31^

Supporting this hypothesis, in our data although it did not face financial barriers, the group with lower screening rate (profile A) less frequently visited a gynecologist than the other women in the CCS population. Also, the difference in regularity of blood tests between the two very healthy profiles A and B may reflect differences in the need to be reassured.

Finally, in the context of the ongoing debate about the benefits and harms of mammography,^39,40^ increased evidence regarding false-positive results or overdiagnosis in BCS possibly leads to a more critical public view and may induce or reinforce, skepticism in women^41^; this may be observed especially among those with high socioeconomic position. Information on women's perception, beliefs, and attitudes to BCS and CCS was not available in our data, but further research is needed to investigate to what extent these factors are associated with BCS and CCS.

Our study is based on a large national sample including a wide spectrum of detailed individual-level information. Moreover, data on primary care utilization and cancer screening were obtained from administrative records removing any reporting bias. In addition, whereas most studies only account for the number of visits to the GP, we included a more comprehensive measure of primary care utilization by considering blood tests.

However, several limitations should be noted. First, CONSTANCES is based on voluntary participation, and participants tend to more frequently be retired, be healthier, have higher socioeconomic position, and visit more regularly GP, dentists, and specialists.^42^ They may therefore be more prone to prevention than nonparticipants. Therefore any selection bias cannot account for the identification of profile A, healthy women with a high socioeconomic position, and little engaged in cancer screening.

Second, the cross-sectional study design makes it improper to infer a causal link between health, primary care utilization, and cancer screening. It is nevertheless unlikely that women's health or primary care utilization radically changed after performing their last cancer screening.

Third, we had to exclude women with missing data, which may cause bias. Indeed, women with missing data had less favorable socioeconomic circumstances, worse health, and poorer primary care utilization than those with completed data.

## Conclusion

Our results suggest that primary care utilization contributes to the adherence to BCS and CCS in close relation with women's health. Policies aiming at increasing cancer screening participation should simultaneously account for these characteristics and consider more specific groups than what is usually done. Our results also stress the need to improve GP training regarding how to communicate about cancer screening with their patients. Overall, our results could inform policy makers and health professionals regarding the groups of women who do not meet the screening recommendations and should be particularly targeted. Although we identified profiles associated with decreased cancer screening participation, these profiles are likely to reflect women's attitude toward prevention more generally.

## Abbreviations Used

BCS: breast cancer screening
BMI: body mass index
CI: confidence interval
CCS: cervical cancer screening
CONSTANCES: Cohort of people visiting Health Screening Centers
GP: general practitioner
OR: odds ratio
